# Safety and Efficacy of Ixekizumab and Antiviral Treatment for Patients with COVID-19: A structured summary of a study protocol for a Pilot Randomized Controlled Trial

**DOI:** 10.1186/s13063-020-04925-8

**Published:** 2020-12-04

**Authors:** Panpan Liu, Zhijun Huang, Mingzhu Yin, Chun Liu, Xiang Chen, Pinhua Pan, Yehong Kuang

**Affiliations:** 1 The Department of Dermatology, Xiangya Hospital, Central South University, Changsha, China; 2 Hunan Key Laboratory of Skin Cancer and Psoriasis, Changsha, China; 3 Hunan Engineering Research Center of Skin Health and Disease, Xiangya Hospital, Central South University, 87 XiangYa Road, Changsha, 410008 Hunan China; 4 Center for Clinical Pharmacology, The Third Xiangya Hospital, Central South University, Changsha, China; 5 Department of Respiratory and Critical Care Medicine, The Third Xiangya Hospital, Central South University, Changsha, China; 6 Gerontology Center of Xiangya Hospital, Central South University, Changsha, China; 7 The Respiratory Department, Xiangya Hospital, Central South University, 87 XiangYa Road, Changsha, 410008 Hunan China

**Keywords:** COVID-19, Randomized controlled trial, protocol, Ixekizumab, IL-17A, Safety, Efficacy

## Abstract

**Objectives:**

A severe epidemic of COVID-19 has broken out in China and has become a major global public health event. We focus on the Acute Respiratory Distress Syndrome (ARDS)-like changes and overactivation of Th17 cells (these produce cytokines) in patients with COVID-19. We aim to explore the safety and efficacy of ixekizumab (an injectable drug for the treatment of autoimmune diseases) to prevent organ injury caused by the immune response to COVID-19. Ixekizumab is a human monoclonal antibody that binds to interleukin-17A and inhibits the release of pro-inflammatory cytokines and chemokines.

**Trial design:**

The experiment is divided into two stages. In the first stage, the open trial, 3 patients with COVID-19 are treated with ixekizumab, and the safety and efficacy are observed for 7 days. In the second stage, 40 patients with COVID-19 are randomly divided into two groups at 1:1 for 14 days. This is a two-center, open-label, randomized controlled pilot trial with 2-arm parallel group design (1:1 ratio).

**Participants:**

Patients with COVID-19 aged 18-75 with increased Interleukin (IL)-6 levels will be enrolled, but patients with severe infections requiring intensive care will be excluded. The trial will be undertaken in two centers. The first stage is carried out in Xiangya Hospital of Central South University, and the second stage is carried out simultaneously in the Third Xiangya Hospital of Central South University.

**Intervention and comparator:**

In the first stage, three subjects are given ixekizumab (“Taltz”) (80 mg/ml, 160 mg as a single hypodermic injection) and antiviral therapy (α-interferon (administer 5 million U by aerosol inhalation twice daily), lopinavir/ritonavir (administer 100mg by mouth twice daily, for the course of therapy no more than 10 days), chloroquine (administer 500mg by mouth twice daily, for the course of therapy no more than 10 days), ribavirin (administer 500mg by intravenous injection two to three times a day, for the course of therapy no more than 10 days), or arbidol (administer 200mg by mouth three times a day, for the course of therapy no more than 10 days), but not more than 3 types). The treatment course of the first stage is 7 days.

In the second stage, 40 randomized patients will receive the following treatments--Group 1: ixekizumab (80 mg/ml, 160 mg as a single hypodermic injection) with antiviral therapy (the same scheme as in the first stage); Group 2: antiviral therapy alone (the same scheme as in the first stage). The length of the second treatment course is 14 days.

**Main outcomes:**

The primary outcome is a change in pulmonary CT severity score (an imaging tool for assessing COVID-19, which scores on the basis of all abnormal areas involved). Pulmonary CT severity score is assessed on the 7th day, 14th day, or at discharge.

**Randomisation:**

In the second stage, 40 patients with COVID-19 are randomly divided into two groups at 1:1 for 14 days. The eLite random system of Nanjing Medical University is used for randomization.

**Blinding (masking):**

The main efficacy indicator, the CT results, will be evaluated by the third-party blinded and independent research team.

**Numbers to be randomised (sample size):**

In the second stage, 40 patients with COVID-19 are randomly divided into two groups at 1:1 for 14 days.

**Trial Status:**

Trial registration number is ChiCTR2000030703 (version 1.7 as of March 19, 2020). The recruitment is ongoing, and the date recruitment was initiated in June 2020. The anticipated date of the end of data collection is June 2021.

**Trial registration:**

The name of the trial register is the Chinese Clinical Trial Registry. The trial registration number is ChiCTR2000030703 (http://www.chictr.org.cn/). The date of trial registration is 10 March 2020.

**Full protocol:**

The full protocol is attached as an additional file, accessible from the Trials website (Additional file 1). In the interest of expediting the dissemination of this material, the familiar formatting has been eliminated; this Letter serves as a summary of the key elements of the full protocol. The study protocol has been reported in accordance with the Standard Protocol Items: Recommendations for Clinical Interventional Trials (SPIRIT) guidelines (Additional file 2).

## Supplementary Information

**Additional file 1.** Full Study Protocol.

**Additional file 2.** Reporting checklist for protocol of a clinical trial.

## Data Availability

The data will be available from the author on reasonable request. E-mail: yh_927@126.com.

