# Supplementary material for: Safety and Efficacy of Ixekizumab and Antiviral Treatment for Patients with COVID-19: A structured summary of a study protocol for a Pilot Randomized Controlled Trial
Source: Trials. 2020 Dec 4;21:999. doi: 10.1186/s13063-020-04925-8 (PMC7716285; doi:10.1186/s13063-020-04925-8)
Supplement: Supplementary file 1 — Additional file 1. Full Study Protocol. [file 13063_2020_4925_MOESM1_ESM.docx]

**Safety and Efficacy of Ixekizumab and Antiviral Treatment for Patients with COVID-19: A structured summary of a study protocol for a Pilot Randomized Controlled Trial**

**Authors:** Panpan Liu^1,2,3†^, Zhijun Huang^4†^, Mingzhu Yin^1,2,3^, Chun Liu^5^, Xiang Chen^1,2,3,6^, Pinhua Pan^7^*, Yehong Kuang^1,2,3^*

**Affiliations:**

^1^ The Department of Dermatology, Xiangya Hospital, Central South University.

^2^ Hunan Key Laboratory of Skin Cancer and Psoriasis.

^3^ Hunan Engineering Research Center of Skin Health and Disease.

^4^ Center for Clinical Pharmacology, The Third Xiangya Hospital, Central South University, Changsha, China.

^5^ Department of Respiratory and Critical Care Medicine, The Third Xiangya Hospital, Central South University, Changsha, China.

^6^ Gerontology Center of Xiangya Hospital, Central South University.

^7^ The Respiratory Department, Xiangya Hospital, Central south university

*Corresponding author.

Corresponding authors at: XiangYa Hospital, Central South University, 87 XiangYa Road, Changsha, Hunan 410008, China. Tel.: +86

13574171102.

Email: Panpan Liu: [liupanpan91@hotmail.com](mailto:liupanpan91@hotmail.com); Zhijun Huang: [huangzj@csu.edu.cn](mailto:huangzj@csu.edu.cn); Mingzhu Yin: [yinmingzhu2008@126.com](mailto:yinmingzhu2008@126.com); Chun Liu: [liuchun7322@163.com](mailto:liuchun7322@163.com); Xiang Chen: [chengxiangck@126.com](mailto:chengxiangck@126.com); Pinhua Pan: pinhuapan668@126.com; Yehong Kuang: [yh_927@126.com](mailto:yh_927@126.com).

^†^These two authors contributed equally to this work.

**Key words:** COVID-19; Randomized controlled trial; protocol; Ixekizumab; IL-17A; Safety; Efficacy

**Manuscript word count: 2804**

**Abstract**

**Introduction**

A severe epidemic of COVID-19 has broken out in China and become a major global public health event. We focus on the ARDS-like changes and overactivation of Th17 cells in patients with COVID-19. To explore the safety and efficacy of ixekizumab against organ injury caused by the immune response to COVID-19.

**Methods and analysis**

The experiment is divided into two stages. In the first stage, the open trial, 3 patients with COVID-19 are treated with ixekizumab, and the safety and efficacy are observed for 7 days. In the second stage, 40 patients with COVID-19 are randomly divided into two groups at 1:1 for 14 days. Patients with COVID-19 aged 18-75 with increased IL-6 levels will be enrolled. Inclusion was initiated in June 2020. The primary outcome is pulmonary CT score, and the secondary outcomes are pulmonary function, clinical symptoms, the incidence of admission to the ICU, the incidence of noninvasive ventilation, 14-day etiological mortality, length of hospitalization, and laboratory examination. The trial will provide data on the efficacy and safety of ixekizumab and antiviral therapy for patients with COVID-19.

**Ethics and dissemination**

The Institutional Review Board of the Xiangya Hospital of Central South University approved the protocol. Results will be disseminated via journal articles.

**Trial registration number**

<http://www.chictr.org.cn/> with code ChiCTR2000030703.

**Strengths and limitations of this study**

This trial protocol first describes a pilot-randomized, evaluator-blind, and controlled clinical trial testing the safety and efficacy of ixekizumab and antiviral treatment in patients with COVID-19.

Possible limitation was that the sample size was not statistically estimated.

**Introduction**

Since December 2019, a serious epidemic of COVID-19 has broken out in China and has become a major global public health event ^[1]^. The World Health Organization officially named the pneumonia epidemic in Wuhan "Corona Virus Disease 2019" (COVID-19). At present, the main source of infection is patients infected by the novel coronavirus. Asymptomatic infection may also be a source of infection. Respiratory droplets and contact transmission are the main routes of transmission. The common signs of coronavirus infection are respiratory symptoms, fever, cough, shortness of breath and dyspnea ^[2]^. In more severe cases, infection can lead to pneumonia, severe acute respiratory syndrome, kidney failure, and even death ^[3]^. At present, there is no specific treatment for diseases caused by novel coronavirus.

In view of the new coronavirus (COVID-19), the researchers analyzed the clinical characteristics of patients with COVID-19 and concluded that the lymphocyte count of patients, especially those with severe pneumonia, decreased significantly, while many inflammatory factors (such as IL-6, TNF-α, etc.) increased significantly, resulting in a cytokine storm (cytokine storm), leading to multiple organ failure. This is an important cause of disease progression and death ^[4]^. The Lancet published the results of minimally invasive pathological sampling of the first new type of coronary pneumonia on February 17. The patients' lungs showed diffuse alveolar injury and hyaline membrane formation, consistent with ARDS findings. The decrease in lymphocytes and the increase in highly proinflammatory CCR4+ CCR6+ Th17 cells can be found by flow cytometry. The results of pathological anatomy are of great significance for the treatment of the disease ^[5]^. We focused on the ARDS-like changes and the overactivation of Th17 cells in patients with COVID-19. Th17 cells play a role in the production of interleukin-17 (IL-17A). At present, research has revealed that the IL-17A/IL-22 axis plays an important role in maintaining the homeostasis of mucous membranes and vascular endothelial cells. Abnormal IL-17A signal transduction can lead to an excessive inflammatory response and cause acute lung injury, such as ARDS ^[6]^. Some studies have shown that the storm of cytokines in IL-17RA knockout mice is greatly weakened after infection with influenza virus. The absence of IL-17RA signaling in this model reduces pulmonary inflammation, weight loss and mortality, indicating that treatment for a variety of inflammatory mediators (including IL-17A) involved in cytokine storm early induction can effectively avoid cytokine storm mortality ^[7]^. However, the importance of Th17 cell and IL-17A participation in cytokine storm in patients with COVID-19 is still unclear. It is still unknown whether IL-17A antagonists can be used in the treatment of cytokine storm in patients with COVID-19.

Ixekizumab is a new biological agent targeting IL-17A and has the characteristics of a high clearance rate in the treatment of psoriasis ^[8]^. At the same time, its IgG4-based structure has a weak affinity for Fc γ R, and its ability to trigger antibody-dependent cell-mediated cytotoxicity (ADCC) and complement-dependent cytotoxicity (CDC) inflammatory reactions is also low ^[9]^. Based on the above findings, this study intends to explore the safety and efficacy of ixekizumab against organ injury caused by the immune response to COVID-19.

**Methods**

**Study Design**

The experiment is divided into two stages. In the first stage, the open trial, 3 patients with COVID-19 are treated with ixekizumab, and the safety and efficacy are observed for 7 days. In the second stage, 40 patients with COVID-19 are randomly divided into two groups at 1:1 for 14 days. The results of the first phase will be reported to the ethics committee for evaluation and approval, and then, the second phase of the study will be carried out. Screening for inclusion was initiated on June 2020.

**Eligibility Criteria**

The inclusion criteria for the trial are as follows: the age is 18-75 years old, regardless of sex; COVID-19 will be diagnosed by RT-PCR or gene sequencing; the clinical types are common type (including severe high-risk factors) (multiple lesions of both lungs, or lung imaging showing that the lesion progressed more than 50% within 24 to 48 hours) and severe type; an increase in IL-6 levels; and the patient voluntarily signing the informed consent form.

The exclusion criteria for the trial are as follows: severe new pneumonia infection with one of the following conditions: respiratory failure requiring mechanical ventilation, shock, ICU monitoring and treatment with other organ failure; allergy to ixekizumab or antiviral drugs; ALT/AST>5 times the ULN, neutrophils<0.5 × 10^9/^L, or platelet less than 50 × 10^9^/L; patients with impaired renal function: serum creatinine ≥2 mg/dl (176 μmol/l); complications with severe underlying diseases or a life expectancy of less than 3 months; patients with active hepatitis, definite severe bacterial or fungal infections, tuberculosis infection, or HIV infection; malignant tumors, such as lymphoproliferative diseases; active Crohn's disease; pregnant or lactating women; participation in other intervention clinical trials in the past 3 months; or other situations in which the researchers believe that it is not appropriate to join the group.

The termination standards for the trial are as follows: allergy to research drugs; judgment of the researchers that it is necessary to terminate the study ahead of time because continuing to participate in the study may bring unacceptable risks to the subjects' health based on the occurrence of serious adverse events, or the subjects are unwilling to continue to participate in the study because of the adverse events; the observation of the curative effect was affected by other diseases such as stroke in the trial; or the ethics committee or regulatory body requested that the study be suspended.

The exit standard for the trial indicates that subjects can drop out of the experiment at any time for the following reasons: the subjects withdraw their informed consent; the subjects are lost to follow-up; and the subjects ask to stop or change the medicine. During the study, when the subjects meet the above termination/withdrawal criteria, the researchers can terminate the participation of the subjects and continue the follow-up treatment and follow-up as far as possible according to the medical routine. The reason and date of the subject's withdrawal must be recorded in the case report form and the original data.

**Research center**

This experiment adopts a multicenter design. The first stage is carried out in Xiangya Hospital of Central South University, and the second phase is carried out simultaneously in the third Xiangya Hospital of Central South University.

**Randomization**

The first stage is an open design. In the second stage, central randomization is used, and the eLite random system of Nanjing Medical University is used. The main efficacy indicator CT results will be evaluated by the third-party blind method outside the research team.

**Baseline Procedures**

The following assessments must be completed during the screening period: medical history, occupational history, drug history, hobbies and smoking history; vital signs: respiratory rate, heart rate, and blood pressure; clinical symptom evaluation: dyspnea score; routine laboratory examination: routine blood tests, routine urine tests, liver and kidney function, blood glucose, electrolytes, troponin, serum ferritin, CRP, IL-6, TNF-α, PCT and T cell subsets; arterial blood gas analysis: pH, PaO2, PaCO2 and oxygenation index; routine electrocardiographic examination (ECG); chest high-resolution CT (HRCT) examination and score; and APACHE II score.

**Enrollment and follow-up**

Those who meet the enrollment criteria will enter the intervention:

On the 3rd day of enrollment, the following procedures will be performed: (1) record concomitant medication; (2) record adverse events; and (3) carry out CT examination.

On the 7th day (±1 day)), the following procedures will occur: (1) record the combined use of drugs; (2) record adverse events; (3) measure vital signs: respiratory rate, heart rate and blood pressure; (4) evaluate clinical symptoms: dyspnea score; (5) perform routine laboratory examination: routine blood, routine urine, liver and kidney function, blood glucose, electrolyte, troponin, hypersensitive CRP, procalcitonin, serum ferritin; (6) assess inflammation index (CRP, IL-6, TNF- α), infection index (routine blood tests, PCT) and immune function (T cell subsets); (7) analyze arterial blood gas: pH, PaO2, PaCO2 and oxygenation index; (8) perform HRCT examination and score; and (9) calculate APACHE II score.

Fourteen days after admission (±3 days) or discharge, (1) record the combined use of drugs; (2) monitor possible adverse reactions to ixekizumab; (3) check vital signs: respiratory rate, heart rate, blood pressure; (4) evaluate clinical symptom: dyspnea score; (5) perform routine laboratory examination: routine blood, routine urine, liver and kidney function, blood glucose, electrolyte, troponin, hypersensitive CRP, procalcitonin, serum ferritin; (6) assess inflammation index (CRP, IL-6, TNF-α), infection index (routine blood, PCT) and immune function (T cell subsets), and so on; (7) analyze arterial blood gas: pH, PaO2, PaCO2 and oxygenation index; and (8) perform HRCT examination and score; (9) APACHE Ⅱ score was calculated (Figure 1).

**Treatment**

**Drugs**

Ixekizumab (*Eli Lilly and Company*): 80 mg/ml, 160 mg as a single hypodermic injection.

Antiviral treatment: according to the diagnosis and treatment plan for pneumonia related to infection by novel coronavirus of the Health Commission (trial version 6 and update).

**Treatment Protocols**

After the subjects sign the informed consent form, the researchers will complete the evaluation within 2 days.

The first phase: open design. Three subjects are enrolled in the same center. They are given ixekizumab and antiviral therapy (aerosol inhalation of α-interferon, lopinavir/ritonavir, chloroquine, ribavirin, or arbidol but not more than 3 types). The vital signs and changes in disease, inflammation indexes (CRP, IL-6, TNF-α) and infection indexes (routine blood tests) are monitored. Changes in PCT and immune function (T cell subsets) are detected. If there are no serious drug-related safety events in the patient's condition, the results of the first stage will be reported to the ethics committee for evaluation and approval, and the second phase of the study will be carried out.

In the second stage, 40 subjects are randomly divided into two groups at a 1:1 ratio. They are given ixekizumab and antiviral therapy (aerosol inhalation of α-interferon, lopinavir/ritonavir, chloroquine, ribavirin, or arbidol but not more than 3 types) or antiviral therapy alone. The course of treatment is 14 days. The vital signs, blood oxygen saturation, CT and other related indexes are followed up on days 0, 3, 7, and 14. The changes in inflammation indexes (CRP, IL-6, TNF-α), infection indexes (blood routine, PCT) and immune function (T cell subsets) and other indexes are monitored. The last follow-up is completed on the 14th day or early recovery and discharge (Figure 2).

**Cointerventions**

**Permissible combination**

According to the diagnosis and treatment program of pneumonia related to infection by the novel coronavirus of the Health Commission (trial version 5), antiviral treatment, nutritional support, maintenance of internal environmental stability and necessary antibiotics can be combined. If the condition requires short-term use of glucocorticoids, it is accurately recorded.

**Prohibited combinations**

Other drugs that regulate the storm of immune factors, such as IL-6R monoclonal antibodies or TNF-α inhibitors, may not be used. It should also not be used at the same time as live vaccines.

**Outcomes and Measures**

**Primary outcomes**

Changes in pulmonary CT score (7th day, 14th day or discharge time). The third-party imaging experts invited by the project team will carry out the blind evaluation.

**Secondary outcomes**

1) Pulmonary function prognostic indicators: percentage of subjects reporting each severity level (on a 7-point scale) (https://clinicaltrials.gov/ct2/show/NCT04280705). The scale is the assessment of clinical status at the first evaluation on a specified study day. The indicators are as follows: (1) death; (2) admission due to invasive mechanical ventilation or extracorporeal membrane oxygenation (ECMO); (3) hospitalization with noninvasive ventilation or high-flow oxygen equipment; (4) hospitalization requiring oxygen inhalation; (5) hospitalization without oxygen inhalation; (6) hospital discharge with limited activity; and (7) no need to be hospitalized and activities are not restricted.

2) Clinical symptom relief time: Clinical symptom relief is defined as normal body temperature for >72 hours (without taking antipyretic drugs or glucocorticoids) and no conscious dyspnea or relief of dyspnea.

3) The incidence of admission to the ICU on the 3rd day, 7th day and 14th day;

4) The incidence of noninvasive ventilation on the 3rd, 7th and 14th days;

5) 14-day etiological mortality and 14-day all-cause mortality;

6) Length of hospitalization;

7) Changes in troponin, inflammation indexes (CRP, IL-6, TNF-α), infection indexes (blood routine, PCT) and immune function (T cell subsets)) on the 7th and 14th days.

8) The ratio of arterial partial pressure of oxygen (PaO2) to fraction of oxygen inhalation (FiO2) on the 3rd, 7th and 14th days.

**Security assessment**

**Safety index**

The incidence of adverse events/serious adverse events; laboratory examination (blood routine, blood biochemistry, urine routine), vital signs, physical examination and electrocardiogram before and after treatment.

**Report and follow-up of adverse events and serious adverse events.**

Adverse events and serious adverse events should be diagnosed clinically as much as possible, and symptoms and signs can only be used if the diagnosis is unknown. Diagnostic terms can be referred to the WHO glossary. In the case of bacterial or fungal infection, active treatment will be given according to clinical routine and novel coronavirus diagnosis and treatment and followed up until cured or stable.

**Data Collection and Data Management**

In this study, the team of researchers is responsible for data management to ensure the authenticity, integrity, privacy and traceability of the clinical trial data. Two people will enter clinical data into the Epidata3.0 database. This study adopts the method of manual verification, and professional statisticians are invited to verify the data if necessary. After the data are entered into the database, if there are any illogical data, it will trigger doubt. These questions need to be reviewed and answered by the researcher or a person authorized by the researcher. Before locking the database, the data administrator should ensure that all queries have been resolved to ensure the completeness and accuracy of the subjects' data. Past medical history and adverse events will be coded according to the MedDRA dictionary, and accompanying medication will be coded using the World Health Organization Drug Dictionary (WHO DD), all of which are in a recognized version.

**Statistical analysis plan**

**Sample Size Projections**

Forty-three cases were planned to be included in the group. Sample size estimation: This study is an open exploratory study, so the sample size is not statistically estimated. According to the estimation of clinical researchers, 43 cases are planned to be included in the group.

The statistical analysis in this study will be performed using SAS software. The statistical description of the quantitative variable calculates the mean, standard deviation, median, 25% quantile Q1, 75% quantile Q3, minimum and maximum.

**Subject distribution**

The screening of the subjects, joining of the groups, completion of the study and reasons for the failure of screening, reasons for early withdrawal, and so on will be summarized and described. The case distribution of each data set, elimination of each analysis set and reasons for elimination, as well as violations of at least one important scheme, will be summarized and described.

**Demographic data and baseline characteristics**

Demographics and baselines will be analyzed in the FAS set and the PPS population. All demographic variables and baseline characteristics (such as age, sex, height, weight, past diseases, past medication, past treatment history, vital signs, etc.) will be summarized. Descriptive statistics (number of subjects, average, standard deviation, minimum, Q1, median, Q3 and maximum) will be used as continuous variables, and classified variables will be used to calculate frequency and percentage ratio.

**Analysis of curative effect**

For the subjects who participated in the study, the FAS set and PPS set will be used to analyze the effectiveness, in which the PPS set is the main analysis set of efficacy analyses.

**Security Analysis.**

Safety evaluation analysis: Safety indicators include adverse events/important adverse events/serious adverse events, laboratory examination, vital signs, physical examination and electrocardiogram and other clinically significant changes. According to the cases of adverse events that occur during the SOC/PT summary trial, the cases and incidence of adverse events between the two groups will be summarized and compared. Descriptive statistical laboratory examination and other indicators, if necessary, compare the changes between the two groups and describe the abnormal cases before and after treatment.

**Interim analysis**

This study will conduct a mid-term analysis by the Data and Safety Monitoring Board (DSMB) to evaluate the dose, efficacy, safety and sample size of the drugs studied.

**Conclusion**

The trial will provide data on the efficacy and safety of ixekizumab and antiviral therapy for patients with COVID-19. The results of this study may help clinicians and policy makers develop safe and effective treatment plans for patients with COVID-19 and reduce mortality.

**Public and patient involvement**

Patients with COVID-19 were not involved in the initial phases of the study.

**Ethics and dissemination**

The Institutional Review Board of the Xiangya Hospital of Central South University approved the protocol on 9 March 2020. The trial was registered at http://www.chictr.org.cn/with code ChiCTR2000030703. Informed consent will be obtained from all the patients enrolled following local regulations. Results will be disseminated via journal articles and communicated to the public by a press release.

**References**

1. Tian H, et al. An investigation of transmission control measures during the first 50 days of the COVID-19 epidemic in China. *Science.* 2020.

2. Cao J, et al. Clinical Features and Short-term Outcomes of 102 Patients with Corona Virus Disease 2019 in Wuhan, China. *Clin Infect Dis.* 2020.

3. Tang X, et al. Comparison of Hospitalized Patients with Acute Respiratory Distress Syndrome Caused by COVID-19 and H1N1. *Chest.* 2020.

4. Huang C, et al. Clinical features of patients infected with 2019 novel coronavirus in Wuhan, China. *Lancet.* 2020;395(10223):497-506.

5. Xu Z, et al. Pathological findings of COVID-19 associated with acute respiratory distress syndrome. *Lancet Respir Med.* 2020.

6. Gurczynski SJ, and Moore BB. IL-17 in the lung: the good, the bad, and the ugly. *Am J Physiol Lung Cell Mol Physiol.* 2018;314(1):L6-L16.

7. Crowe CR, et al. Critical role of IL-17RA in immunopathology of influenza infection. *J Immunol.* 2009;183(8):5301-10.

8. Paul C. Ixekizumab or secukinumab in psoriasis: what difference does it make? *Br J Dermatol.* 2018;178(5):1003-5.

9. Dumet C, Pottier J, Gouilleux-Gruart V, and Watier H. Insights into the IgG heavy chain engineering patent landscape as applied to IgG4 antibody development. *MAbs.* 2019;11(8):1341-50.

**Figure and Figure Legend**

Figure 1. Schematic diagram of trial


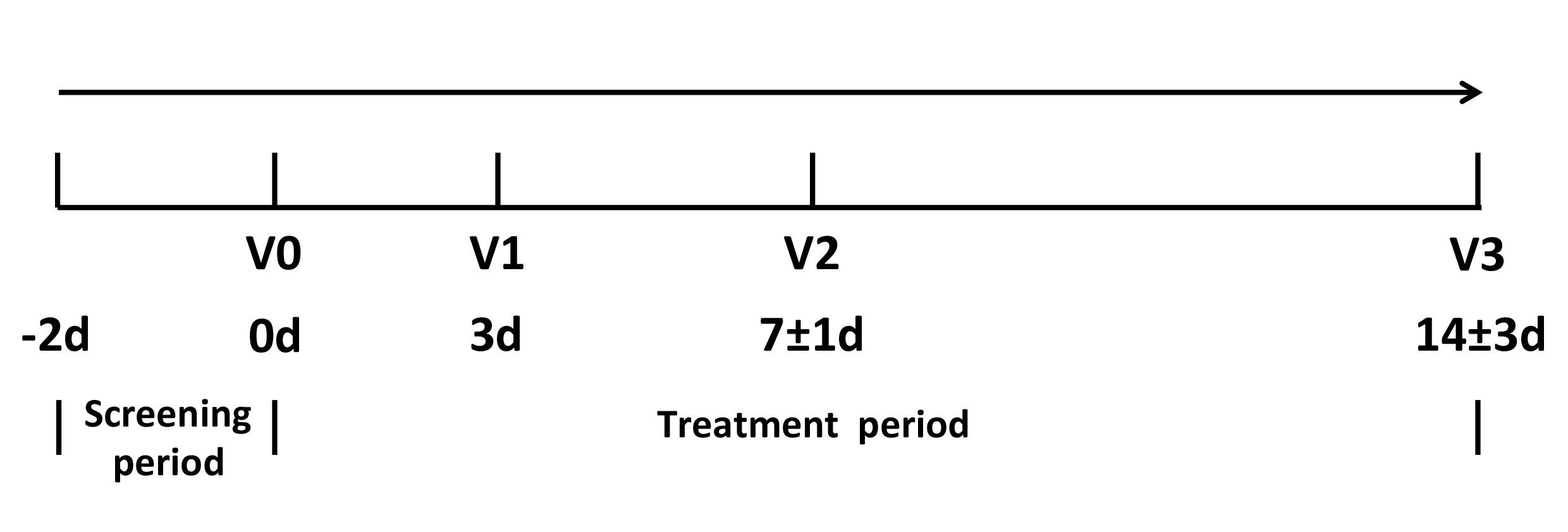


Figure 2. Study Flow Diagram

Assessment the safety and efficacy at day 0, 3, 7, 14

Assessment the safety and efficacy at day 0, 3, 7, 14

Treated with antiviral treatment alone

Treated with ixekizumab and antiviral treatment

Randomization
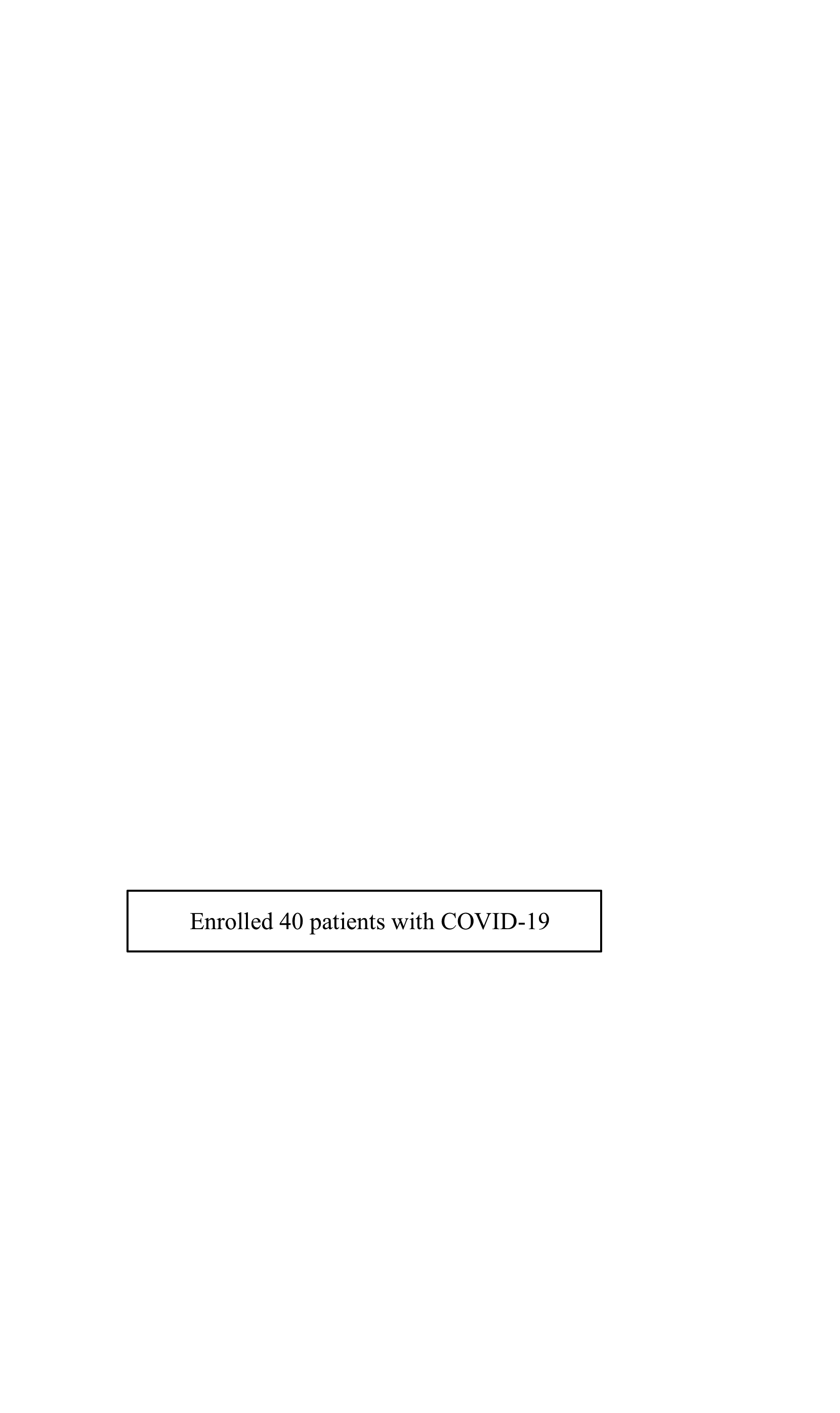
tion

Enrolled 40 patients with COVID-19

Baseline assessment

Referred and informed consent

Assessment the safety and efficacy at day 7

Treated with ixekizumab and antiviral treatment

Enrolled 3 patients with COVID-19

Baseline assessment

Referred and informed consent

Reported to the ethics committee for evaluation and approval

**Contributors**

PL and YK wrote the first draft of the manuscript. The manuscript was critically revisited by YK, MY, CL, and XC. PP and ZH was the primary investigator of the study. YK, PP, ZH, and XC contributed to the study concept, trial design and study protocol. All authors read and approved the final manuscript.

**Funding statement**

This work was supported by the key project of the National Science Foundation (81430075, 81830096), National Natural Science Foundation of China (81573049), Key R & D Program of Hunan Province (2018SK2082), National key R & D program (2018YFC0117004), Emergency Project of Prevention and Control for COVID-19 of Central South University (50270100), and Hunan Provincial Innovation Foundation For Postgraduate (2020zzts251). The sponsors have no role in study design, collection, analysis, interpretation of data, and writing the manuscript.

**Conflict of Interest Disclosures:** None reported.

**Patient and public involvement**

Patients and/or the public were not involved in the design, or conduct, or reporting, or dissemination plans of this research.
